# Commentary: Complex Motor Learning and Police Training: Applied, Cognitive, and Clinical Perspectives

**DOI:** 10.3389/fpsyg.2019.02444

**Published:** 2019-11-01

**Authors:** Mario S. Staller, Swen Körner

**Affiliations:** ^1^Department of Police Science, University of Applied Sciences for Public Administration and Management, Gelsenkirchen, Germany; ^2^Department of Training Pedagogy and Martial Research, German Sport University Cologne, Cologne, Germany

**Keywords:** police, police training, complex motor learning, coaching (performance), ecological dynamics

Di Nota and Huhta (2019) recently provided an informative overview concerning complex motor learning in the context of police training. Reflecting the emphasis on complex motor learning in the context of police training, there are a number of pedagogical practices and coaching decisions which are informed by this paper. As Di Nota and Huhta (2019) explain, that “police trainers generally agree that basic skills training should begin with learning the fundamentals, or component “chunks,” in order to develop proficiency, comfort and safety with a given technique” (p. 10). As such, the authors suggest first learning basic skills and techniques up to a proficient level before further develop expertise “through exposure to increasingly complex and demanding situations” (p. 10) and applying them in performance settings. Concerning this assumption of a linear and modular approach to training (Moy et al., 2015), we raise two issues informed by the ecological dynamics perspective on skill development (Araújo and Davids, 2011; Seifert et al., 2019) that may extend some of the suggestions of Di Nota and Huhta (2019). First, the problem of isolation in complex motor learning and second, the problem of neglecting representative task design before the utilization of complex scenario trainings. It is understandable, that article length restrictions may have impacted on the authors in providing the necessary details that we feel warrants additional mention. Hence, it is not our intention to take away from the quality work presented but rather to support its desired aims.

Firstly, from an applied coaching perspective, the issue of isolating parts of movements in order to integrate them later on in a more complex movement or isolating the movement from the performance context has been observed in the police training domain on various occasions (Cushion, 2018; Staller et al., 2019) and has been met with criticism particularly with regards to transfer (Cushion, 2018; Körner and Staller, 2018; Staller and Körner, in press). The proposition of Di Nota and Huhta (2019) of “chunking” movements in order to “facilitate gaining competence in smaller, more manageable units of information” (p. 2) builds on several implicit assumptions in accordance with Information Processing Theory (Schmidt et al., 2018), that are relevant to the field of policing: (a) isolation of performance components effectively contribute to skill development and transfer to the field, (b) the existence of an ideal movement solution, (c) their repetition with low degrees of variation, and (d) the learning process as a coaching centered endeavor with emphasis on prescriptive instruction and feedback. These assumptions are heavily challenged by the ecological dynamics framework (Araújo et al., 2017; Seifert et al., 2017, 2019), which started to gain traction in the sport (Correia et al., 2018) and policing domain (Körner and Staller, 2018). According to this perspective, skill learning better refers to the process of adapting and attuning to the environment, instead of reproducing an “ideal” technique” out of context (Araújo and Davids, 2011). Adopting this perspective leads to pedagogical approaches that facilitate the emergence of greater functional relationship between the learner, conceptualized as adaptive and self-organized system, and the performance environment instead of pursuing a perfect technique (Körner and Staller, 2018; Renshaw and Chow, 2018). By highlighting the importance of performance-environment coupling, perceptual-action coupling and consideration of the non-linearity of the learning process and performance in the field (Seifert et al., 2019), the ecological dynamics framework offers a useful perspective on skill development and transfer especially with regards to designing practice activities in general (Pinder et al., 2011; Krause et al., 2017) and in the law enforcement domain particularly (Staller et al., 2017; Körner and Staller, 2018). Furthermore, concerning the assumption of an ideal movement as a basis of the linear approach to learning, data has shown, that due to ongoing changes within individual and environmental constraints there is no repetition within a repetition on an intraindividual basis (Schöllhorn, 1999; Schöllhorn et al., 2012) and that movement solutions to specific problems at hand vary between individuals (Dicks et al., 2017). This applies even to non-complex movements (Bernstein, 1967). In the context of police training, the functional role of intra- and interindividual variability has recently been pointed out (Körner and Staller, 2018) and is backed up by current data highlighting the functionality of adaptive behavioral solutions in police self-defense task (Körner et al., 2019). Allowing and promoting functionality and variability of individual solutions for problems at hand is as much a pedagogical challenge for the coach as providing descriptive feedback and an external focus of attention.

The second issue relates to the actual coaching practice within the context of police training. Even though Di Nota and Huhta (2019) complement the need for performance-environment and perceptual-action coupling in learning tasks by referring to scenario-based training as the “gold standard for complex motor learning for police” (p. 10), we would argue, that this approach is too short-sighted for effective skill development in police training. Data from training observations in police training (Cushion, 2018; Staller et al., 2019) showed, that trainings tasks—except for scenario-based training—regularly seem to lack representative task design leading to a lack of transfer in more complex tasks later on. These data indicate that representatively designed tasks are employed in police training, but not in the beginning of training, when practice tasks regularly involve partner interaction on a one-to-one basis. The concept of representative task design (Pinder et al., 2014; Staller et al., 2017) may provide police trainers and coaches with the tools to practice the needed skills in an integrated manner (incorporating perception-action and performance-environment coupling) beyond scenario-based training. Police trainers should generally focus on representatively-designed, high-quality interactions of training partners right from the start (Staller and Körner, 2018) instead of constraining for disintegrated motor learning in the frame of isolated practices out of context in the beginning and only subsequently focusing on representative task design in the context of scenario-based training. This may be achieved by framing partner drills (even in the early stages of training) as representative simulations with clear roles and responsibilities providing the learner with the opportunity to act upon information variables offering situationally and gradually varying demands (physical, cognitive-perceptual, and affective) that are representative of the performance context allowing for the acquisition, emergence and attunement of functional skill. Furthermore, these interactions may also serve as a base unit for the analysis for the efficiency of the training process (Araújo and Davids, 2018) when evaluating police training.

## Author Contributions

MS and SK authors contributed equally to the ideas presented. MS wrote the first draft of the paper. Both authors contributed equally to editing the first draft to its final version.

### Conflict of Interest

The authors declare that the research was conducted in the absence of any commercial or financial relationships that could be construed as a potential conflict of interest.
